# Art-making in a family medicine clerkship: how does it affect medical student empathy?

**DOI:** 10.1186/s12909-014-0247-4

**Published:** 2014-11-28

**Authors:** Jordan S Potash, Julie Y Chen, Cindy LK Lam, Vivian TW Chau

**Affiliations:** Art Therapy Graduate Program, The George Washington University, Washington D.C., USA; Department of Family Medicine and Primary Care and Institute of Medical and Health Sciences Education, Li Ka Shing Faculty of Medicine, The University of Hong Kong, 2/F William MW Mong Block, 21 Sassoon Road, Pokfulam, Hong Kong China; Department of Family Medicine and Primary Care, Li Ka Shing Faculty of Medicine, The University of Hong Kong, Hong Kong, China

**Keywords:** Medical humanities, Empathy, Art, Reflective writing, Family Medicine, Medical student

## Abstract

**Background:**

To provide patient-centred holistic care, doctors must possess good interpersonal and empathic skills. Medical schools traditionally adopt a skills-based approach to such training but creative engagement with the arts has also been effective. A novel arts-based approach may help medical students develop empathic understanding of patients and thus contribute to medical students’ transformative process into compassionate doctors. This study aimed to evaluate the impact of an arts-making workshop on medical student empathy.

**Methods:**

This was a mixed-method quantitative-qualitative study. In the 2011–12 academic year, all 161 third year medical students at the University of Hong Kong were randomly allocated into either an arts-making workshop or a problem-solving workshop during the Family Medicine clerkship according to a centrally-set timetable. Students in the arts-making workshop wrote a poem, created artwork and completed a reflective essay while students in the conventional workshop problem-solved clinical cases and wrote a case commentary. All students who agreed to participate in the study completed a measure of empathy for medical students, the Jefferson Scale of Empathy (JSE) (student version), at the start and end of the clerkship. Quantitative data analysis: Paired t-test and repeated measures ANOVA was used to compare the change within and between groups respectively. Qualitative data analysis: Two researchers independently chose representational narratives based on criteria adapted from art therapy. The final 20 works were agreed upon by consensus and thematically analysed using a grounded theory approach.

**Results:**

The level of empathy declined in both groups over time, but with no statistically significant differences between groups. For JSE items relating to emotional influence on medical decision making, participants in the arts-making workshop changed more than those in the problem-solving workshop. From the qualitative data, students perceived benefits in arts-making, and gained understanding in relation to self, patients, pain and suffering, and the role of the doctor.

**Conclusions:**

Though quantitative findings showed little difference in empathy between groups, arts-making workshop participants gained empathic understanding in four different thematic areas. This workshop also seemed to promote greater self-awareness which may help medical students recognize the potential for emotions to sway judgment. Future art workshops should focus on emotional awareness and regulation.

## Background

Within medical training, some argue that empathy is unnecessary or should be carefully balanced with objectivity and etiquette [1]. Many others feel that empathy complements and facilitates successful clinical consultations and quality of care [2,3]. The debate often revolves around the relative importance that is placed on doctors being astute diagnosticians and treatment providers or whether they should be able to establish meaningful relationships with patients. Given the limited time within medical education, treating patients is often given precedence over attending to patients. Although empathy can be introduced at all stages of a medical career, medical school provides a secure base as medical student empathy predicts future doctor-patient empathy [4]. Alongside the necessary medical training, many medical students see empathy as a desirable trait for themselves [5,6].

In order for empathy to be viewed as an essential component of medicine, medical schools have to ensure adequate time for it in the curriculum [7], include it as a criterion for assessment [8,9], and provide faculty role models [10]. To this end, several studies have demonstrated the effectiveness of teaching students the emotional aspects of pain [11,12]. Karkabi, Cohen and Castel [13] demonstrated that showing fine art paintings that depicted suffering, writing short stories and engaging in discussion helped family doctors achieve a new perspective on their patients. Other creative approaches to teach empathy within medical education include acting [14,15], narrative writing [16], visual journaling [17], poetry [18], and guided museum visits [19,20].

By creating art and poetry in response to patient situations, practitioners can come to a personal and intimate understanding of various illness experiences, as well as, expanded perceptions [21]. Within medical training and professional development, reflections on the arts encourage multiple perspectives that can help to provide insight and awareness as to how a patient experiences pain and suffering [22]. Introducing an arts-based approach to exploring empathic understanding of patients can facilitate medical students’ transformative process into compassionate doctors.

## Methods

This study adopted a mixed method approach to answer the research questions: (1) How does an arts-based activity affect medical student empathy and (2) What other impacts does an arts-based activity have on medical students?

### Ethical approval

Ethical approval was obtained from the Human Research Ethics Committee for Non-Clinical Faculties at The University of Hong Kong (Ref. No. EA150811).

### Participants

The target subjects included all 161 Year 3 medical students who rotated through the Family Medicine (FM) Junior Clerkship at the University of Hong Kong. This clerkship was embedded in the 10-week multidisciplinary teaching block which ran in 3 rotations from October 2011-April 2012. Year 3 medical students were selected for this study because this is the first point during medical studies in which students have regular clinical interactions with patients. Research has also noted the decline of empathy in medical students during this year [7].

### Procedure

Students were randomly assigned into either an arts-making or a clinical problem-solving workshop according to their schedule during the FM curriculum. Each interactive workshop lasted 3 hours, took place in the same venue, and was led by the same pair of facilitators: a FM academic staff member as well as a qualified art therapist. An art therapist was included to encourage expressive art making and to help elicit meaningful reflections. Students submitted an assignment which formed part of the FM continuous assessment. Students attended a single workshop in a group of approximately 25 participants. Data were collected from October 2011 through April 2012, with workshops approximately two-thirds into each rotation in November 2011, January and March 2012.

Students in the arts-making workshop were led in a guided visualization to recall a memory of a time they witnessed a patient in pain or suffering. Afterwards, they recorded colors, smells, sounds, objects, and feelings on separate pieces of paper. By rearranging the words and adding additional ones as necessary, students created a poem to describe the memory. Next, they created a drawing or painting based on the poem. The students displayed their art and poem and discussed them in small and large groups. Students submitted a reflective essay about how the art making experience affected their understanding of patients and themselves.

Students in the clinical problem-solving workshop observed or participated in a role play with one of the facilitators playing the role of the patient. Under the guidance of the facilitators, students then problem-solved the clinical case and discussed their observations with the large group. These students submitted a written extended case commentary discussing a clinical case they had observed with a discussion on how the case demonstrated the principles of patient care in FM.

### Outcome measures

To measure empathy, students completed the Revised Jefferson Scale of Empathy – Student Version (JSE) [23,24] during the initial orientation briefing and at the conclusion of each block. The JSE consisted of 20 statements rated on a 7-point Likert scale (1 = Strongly Disagree,…, 7 = Strongly Agree). A higher JSE score equated to a higher level of empathy. This measure was developed and validated in a US medical school [23] and has been validated in the Asian context among medical and nursing students [25-27]. To explore the impact of the art-making workshop on participants, themes identified from the poetry, art and reflective essays produced by students in the arts-making workshop comprised the outcome measure.

### Data analysis

For the JSE scores descriptive statistics and paired t-test or ANOVA were performed to determine significance between pre-test and post-test, as well as, between the two groups. To further understand the perception of the arts-making workshop in a more in-depth manner, purposive sampling was used to select reflective essays which could best serve to illustrate the process of creating arts regarding pain and suffering [28]. To determine which essays to analyze, two researchers independently selected 20 narratives (combination of poem and art) that best adhered to the criteria of aesthetic quality in art therapy [29]: conveyed personally meaningful experience, images matched the intended experience, and image was completed through intentional use of art materials, colors, lines, shapes, symbols and words. Even though this was not an art therapy workshop, these criteria were appropriate as the arts created were intended for emotional expression, rather than for the production of fine art. The qualitative data were analyzed by using line-by-line coding according to both inductive and deductive themes that were later organized into clusters [29]. The researchers generated an initial code book based on a reading of all of the essays submitted and a more critical review. Following, three researchers independently coded the reflective essays of these 20 narratives and met to revise the codes and arrive at consensus.

## Results

The results demonstrated ways in which the arts-making workshop affected medical student empathy, as well as, additional benefits.

### Demographic

Of the 161 eligible participants, 152 consented to take part in the study and 106 completed both pre- and post- questionnaire measures and submitted all assignments for a participation rate of 70% (106/152) (Table 1). The demographics of the two groups and those selected for qualitative analysis were comparable.

**Table 1:** **Characteristics of study participants: third-year medical students at the University of Hong Kong (2011–2012) (N = 106)**

	**Problem-solving workshop (n = 58)**	**Art workshop (n = 48)**	Art workshop: qualitative analysis (n = 20)*
**Characteristic**	**n (%)**	**n (%)**	**n (%)**
**Gender**			
Male	34 (58.6)	29 (60.4)	10 (52.6)
Female	24 (41.4)	19 (39.6)	9 (47.4)
**Age (years)**			
18-20	21 (36.2)	15 (31.3)	7 (36.8)
21-23	33 (56.9)	28 (58.3)	11 (57.9)
>23	4 (6.9)	5 (10.4)	1 (5.3)
**Ethnicity**			
Chinese	57 (98.3)	48 (100)	19 (100)
South East Asian	1 (1.7)	(−)	(−)
**Route of admission** ^**†**^			
JUPAS	24 (41.4)	16 (33.3)	7 (36.8)
EAS	20 (34.5)	18 (37.5)	6 (31.6)
Others	14 (24.1)	14 (29.2)	6 (31.6)

*Note.* Complete-case analysis was adopted. Because of rounding, the percentages may not add up to a hundred.

*Among the 20 selected, 19 students provided their background information.

^†^The majority of medical students in Hong Kong are admitted directly from local secondary schools through the Joint University Programmes Admissions System (JUPAS) (after Grade 12 equivalent) and Early Admissions Scheme (EAS) (after Grade 11 equivalent). The others are admitted from local Hong Kong international schools/overseas secondary schools or have already completed an undergraduate university degree.

### Quantitative

Descriptive statistics showed decreases in empathy from the beginning to the end of each rotation. There was no difference between the two groups or association of demographic characteristics with empathy. This change was only statistically significant for participants of the arts-making workshop (paired t-test: t(47) =2.57, p <0.05), (Table 2). Further investigation of this finding revealed statistically non-significant patterns for certain JSE items that revealed differences between the two groups. For the 4 out of the 5 questionnaire items which specifically related to the potential “influence” of emotions on medical judgment, participants in the art making workshop had a much sharper decline in JSE score (or much smaller improvement) compared with the other group. For the questionnaire items relating specifically to “empathy”, both groups showed minimal change in scores which were comparable to each other (Table 3).

**Table 2:** **Third year medical students’ Jefferson Scale of Empathy mean scores over time by group (N = 106)**

**Group**	**Pre-test**		**Post-test**		**Group X time**	
	**Mean**	**SD**	**Mean**	**SD**	**F**	***p*** **-value**
**Workshop (n)**						
Art-making (48)*	106.6	12.4	102.2	14.3	2.38	0.13
Problem-solving (58)	107.2	11.5	106.6	14.7		
**Gender (n)**						
Male (63)	107.1	11.9	104.3	15.9	0.24	0.62
Female (43)	106.7	11.9	105.1	12.7		
**Age (years) (n)**						
18-20 (36)	109.8	9.3	106.6	13.6	0.22	0.80
21-23 (61)	105.2	12.4	103.1	15.3		
>23 (9)	107.2	15.9	107.0	13.6		
**Route of admission**						
JUPAS (40)	102.9	10.6	100.4	14.5	0.02	0.98
EAS (38)	109.4	10.5	107.5	13.9		
Others (28)	109.3	13.9	106.7	14.8		

*t(47) =2.57, *p*-value <0.05 by paired t-test.

**Table 3:** **Examples of statements on the revised Jefferson Scale of Empathy (student version) showing differences between groups for questionnaire items pertaining to influence versus items pertaining to empathy**

	**Art-making**			**Problem-solving**		
	**Pre n = 48**	**Post n = 48**	**Difference**	**Pre n = 58**	**Post n = 58**	**Difference**
**Items pertaining to influence**						
1) Physicians' understanding of their patients' feelings and the feelings of their patient's families does not influence medical or surgical treatment.*	5.48	5.13	−0.35	5.72	4.97	−0.76
8) Attentiveness to patient's personal experiences does not influence treatment outcomes.*	5.48	5.11	**−0.37**	5.55	5.43	**−0.12**
11) Patient's illnesses can be cured only by medical or surgical treatment; therefore, physician's emotional ties with their patients do not have a significant influence in medical or surgical treatment.*	5.67	5.06	**−0.61**	5.62	5.53	**−0.09**
14) I believe that emotion has no place in the treatment of medical illness.*	5.88	5.38	**−0.50**	5.81	5.76	**−0.05**
18) Physicians should not allow themselves to be influenced by strong personal bonds between their patients and their family members.*	3.50	3.75	**0.25**	2.98	3.55	**0.57**
**Items pertaining to empathy**						
9) Physicians should try to stand in their patient's shoes when providing care to them.	5.85	5.73	−0.13	5.71	5.57	−0.14
15) Empathy is a therapeutic skill without which the physician's success is limited.	5.50	5.31	−0.19	5.29	5.40	0.10
17) Physicians should try to think like their patients in order to render better care.	5.10	5.02	−0.08	5.07	5.16	0.09
20) I believe that empathy is an important therapeutic factor in medical treatment	5.67	5.67	0.00	5.81	5.90	0.09

*These statements have been reverse scored. Therefore, in this table a higher score equates to a higher level of empathy as if the items were positively worded.

Numbers in bold show large differences between groups.

### Qualitative

The qualitative comments from the reflective essays revealed benefits of the arts-making workshop (Table 4) and empathic understanding gained (Table 5).

**Table 4:** **Summary of qualitative themes: benefits of arts-making workshop (as recorded in reflective essays)**

**Theme**	**Sub-Theme**	**Example**	**Number of participants**
**Reflection**		“reflecting on our own experience and express it”	4
**“Humanistic side”**		“medical students are deprived of opportunities of developing their artistic or humanistic side, and such a chance would help them realize they are not robots.”	4
**Clarity**	*revise previous ideas*	“a great opportunity to revise the scenario in our mind (visual thinking) and get to know the perception of feelings in that scenario.”	2
	*new understanding*	“Many of us, shocked and reluctant at first, eventually had drawn various pieces of artworks that told their own stories… also helped us to learn more on the emotions and pain that patient suffered from.”	5
**“Enlightening”**		“enlightened us”	3
**Relaxing**		“chance to escape from the highly-intensive lessons and relax a bit”	5
**Develop creativity and imagination**		“It encouraged a completely different and unique thinking process – through the use of art and creative thinking”	2

**Table 5:** **Summary of qualitative themes: empathic understanding gained (as recorded in reflective essays)**

**Theme**	**Sub-theme**	**Example**	**Number of participants**
**Self-awareness**	*general awareness*	“Apart from learning about patients and the way to communicate with patients, I also understood more about myself.”	5
	*recognizing own feelings*	“They were scenes in my life that I had probably neglected… I learnt to examine my own emotion at the particular time in a more detailed manner.”	3
	*coping with feelings*	“We need to strike a balance between the feelings, care and let go; otherwise we and the people surrounding us would be the victims of those feelings.”	3
	*realizing limitations*	“I realized how inadequate I am in self-understanding…”	4
	*existential concerns*	“the meaning of life and death issue. It’s such a huge topic for us students to think about, but still we got a few points of view on this session, some of which had never come to my mind before.”	3
**Patient awareness**	*empathy*	“put us into patients’ shoes,”	10
	*holistic/patient-centered care*	“not treating the disease, but more important is that, we are treating the patient himself.”	12
**Understanding pain and suffering**	*awareness of presence*	“Pain and suffering are two feelings that we all feared of sharing. Yet already as a medical student, I have experienced it at close intervals from my dealings with the sick and ill. I have kept it in, locked, ignored and neglected so I can continue my medical duties and tasks as expected by the society.”	5
	*expanded view of pain and suffering*	“I cannot appreciate all the pieces and put myself into the emotion described. I can only appreciate that pain and suffering do exist in many different forms.”	3
	*duality of pain and suffering*	“what is most important it’s the unexpected ‘side effects’ come along, for instance, regaining family bonding.”	3
	*distinguish between physical pain and metal suffering*	“we can explain all those pain through physiological pathway[s],… However, for the mental aspect, it is much more complicated and difficult to understand.”	4
**Role of the doctor**	*alleviate emotional pain*	“In addition to serving as ‘experts’ to provide curative treatment, doctors could also serve as someone who has witnessed multiple similar events to offer support, counseling and a pair of listening ears, which form an integral part of patient management and disease outcome.”	4
	*alleviate physical pain*	“When we encounter different patient, we can give different kinds of medication to relieve their symptoms”	1
	*contribute to pain*	“I told her about the results. She was quite shocked”	1

### Benefits of arts-making workshop

The workshop provided a space for *reflection* to develop students’ “*humanistic side*”. Perhaps what added to this element was that the workshop fostered *clarity* by prompting them to *revise previous ideas* and to achieve *new understanding*. Having an “*enlightening*” experience resulted from the opportunity, “to sit back and ponder… to calm ourselves down”. Some commented that the workshop was also *relaxing*, a change from their regular routine, that allowed them to *develop creativity and imagination* that could be generally applied:

“It had never occurred to me that there was so much room for imagination and creativity in thinking of a person, patient or illness. It occurred to me that there’s no such thing as a black and white world – but rather the difference in our perceptions”.

### Empathic understanding gained

Understanding gained from the workshop related to self, patients, pain and suffering and the doctor’s role. Self-awareness included *general awareness*, but also specifically *recognizing own feelings* and *coping with feelings*. Students described *realizing limitations* and their own need to develop more accurate self-understanding. Another form of self-understanding was gaining awareness on *existential concerns*, such as identity (“what would I be if I am the one sitting on a wheel chair?”) and death (“there’s one thing for sure that death is what all of the lives will encounter and what we should face with love, understanding and sincerity”).

**Figure 1:**
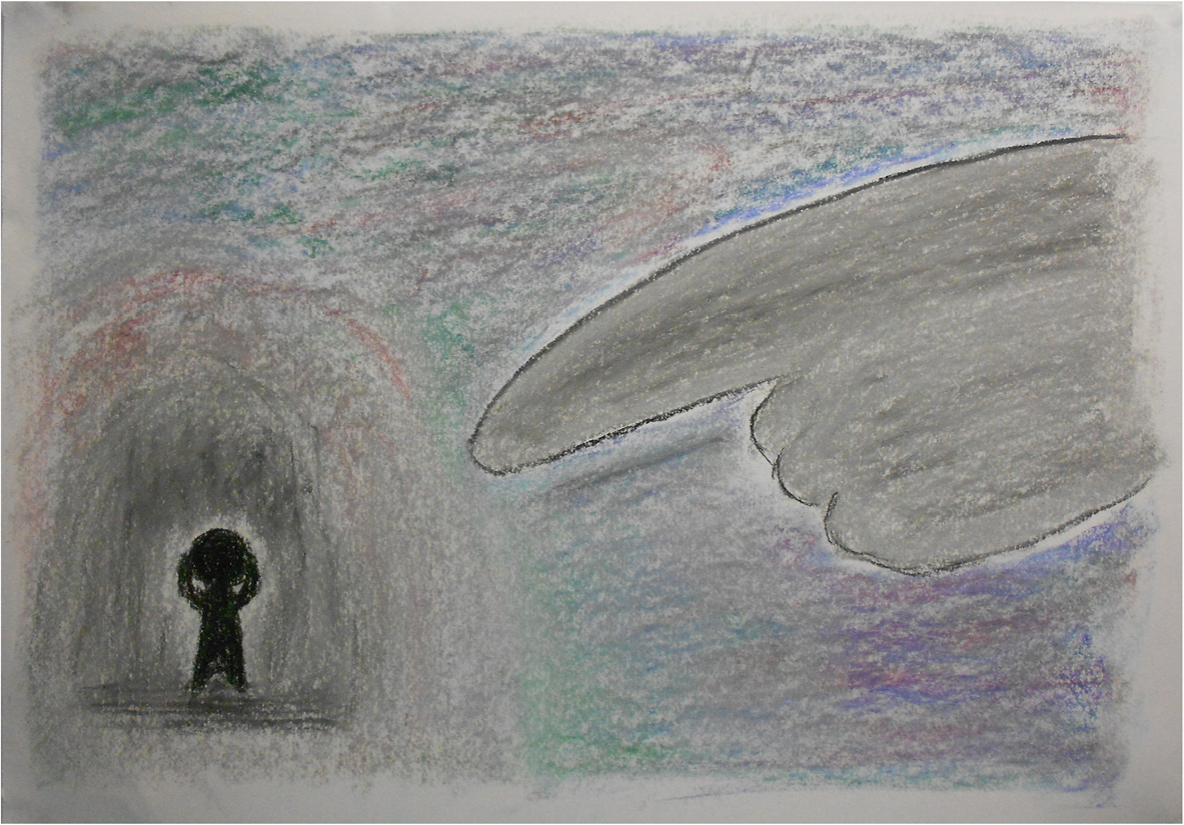
**Finger pointing.**

Increased understanding of patients included *empathy*, which was described as adopting the “patients’ perspectives”, and “strongly feel[ing] his insecurities and desire”. Students expressed appreciation for *holistic/patient-centered care* in writing, “If we just focus on the diseases, we can never get to know the pain and suffering that patients encounter”. Figure 1 is a drawing that shows one student’s attempt to look beyond the patient’s presenting problem, “If I interpret this sentence just based on wordings alone, I would have missed the sense of guilt she was hiding deep inside her heart”.

**Figure 2:**
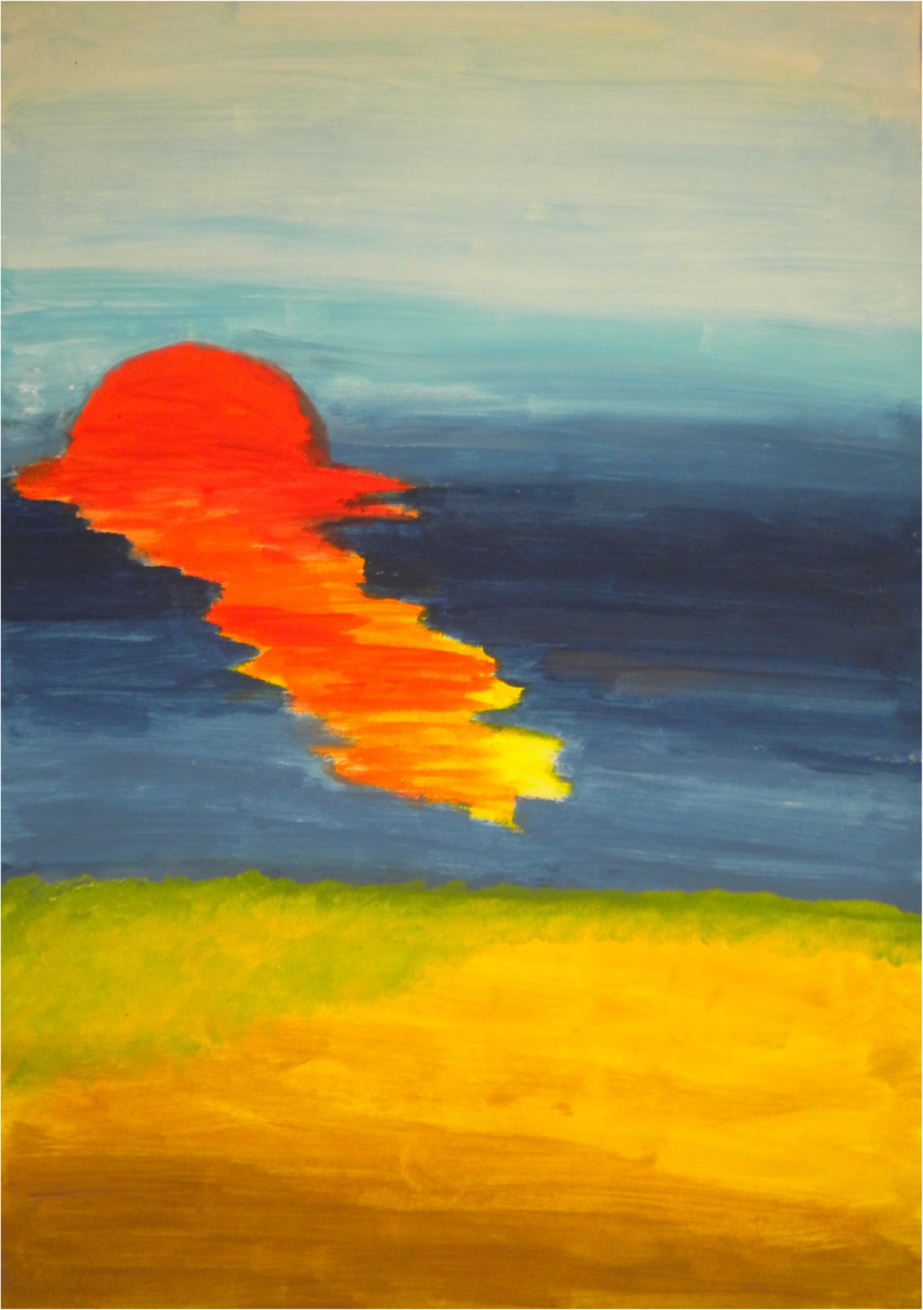
**Sunrise sunset.**

Understanding pain and suffering began with *awareness of [its] presence* after having “kept it in, locked, ignored and neglected so I can continue my medical duties and tasks as expected by the society”. There was an *expanded view of pain and suffering*, as well as, accepting the *duality of pain and suffering* that relate to unexpected benefits that derive from illness. Figure 2 depicts this dichotomy by inviting the viewer to imagine both a sunrise and sunset simultaneously. Such understanding prompted students to *distinguish between physical pain and mental suffering*, as one student wrote:

“Disease can harm your physical body, it can also take away your soul. After the operation, the wound… will eventually fade out. But if the operation was done on you without any social support and understanding, or your soul has already been taken away, that is true suffering”.

Lastly, understanding the role of the doctor revealed the potential to *contribute* and *alleviate* both *physical* and *emotional pain*. One comment that summarized these ideas was:

“In addition to serving as ‘experts’ to provide curative treatment, doctors could also serve as someone who has witnessed multiple similar events to offer support, counseling and a pair of listening ears, which form an integral part of patient management and disease outcome”.

## Discussion

The study demonstrated promise of arts-making engagement for medical students’ emotional learning in Hong Kong. Arts-making provided a novel approach to students who are accustomed to traditional teaching topics and formats. Achieving both reflection and relaxation demonstrated that serious study can take place in a creative atmosphere that has the potential for intense learning about oneself, others and the world. To complement the existing medical curriculum, the arts teach empathy [14], make for more engaged learning [15], and reduce stress [17]. This research additionally demonstrated that art-making can foster a relaxing atmosphere in which meaningful reflection and personal development can occur.

This research offered mixed findings on the role of this workshop for enhancing empathy. The decrease in empathy as measured by the JSE during the clinical year was consistent with prevailing research [30]. The significant decrease for participants in the arts-making workshop was surprising given the findings from the qualitative data that revealed the opposite. One possible reason for the seemingly contradictory finding could be that the JSE was not valid or sensitive enough in this setting. Though the instrument has been shown to have good psychometric properties among comparable Asian populations, it had not been formally validated in Hong Kong. Though the students attended an English medium university and are proficient in English, for many English is a second language. Students’ ability to accurately answer some of the questionnaire items, particularly the negatively worded ones, may have been affected by their English fluency [31]. Additionally as medical education in Hong Kong occurs at the bachelor’s level, the majority of medical students are admitted directly from high school. Their young age and corresponding level of maturity may reflect a still-developing understanding of “empathy”.

While these rationales should have been true for all students, there may have been differences regarding the nature of the two groups. The students in the arts-making workshop were asked to direct their attention to a specific patient, whereas those in the problem-solving group focused on an imaginary case example. In addition, the JSE asks about general scenarios, instead of specific encounters. Perhaps further workshops are needed to help students understand how empathy for an individual patient might be transferred to all patients. Additionally, students in the arts-making workshop were focused on their own emotional self-awareness when confronted with pain and suffering, rather than fully adopting the patient’s perspective. This may have affected their scores on the JSE as well. This last idea is supported by the differences in the JSE scores between the two groups suggesting the idea that the arts-making workshop affected emotional awareness. This is revealed in the pattern for items on the JSE that described the influence of emotion in medical decision making. A student in the art-making workshop summarized this sentiment,

“However, it will not be helpful if we are too emotionally charged. After all, if we easily feel depressed because of a patient’s condition, how are we to offer another point of view for them?”

The increased self-awareness of emotional states as a result of the arts-making workshop may have made students more cognizant of how their rational judgment could be influenced by emotion. Those in the problem-solving workshop may not have come to this conclusion, as evidenced by the relatively stable answers on the JSE. Perhaps the greater score change in the JSE items that relate to emotional influence on medical decision-making demonstrated an awareness that participants of the arts-making workshop obtained and the realization that physician emotions can affect medical treatment. Although the students may have reacted to these emotions by answering that the physician emotions should be limited in patient care, in fact, this may be an overreaction related to uncertainty as to how to best cope with emotions when they present themselves. Perhaps, it would be beneficial to focus on self-awareness as means to helping students learn that emotions and rationality co-exist in medicine as there is a time for taking the patient’s subjective view, while also maintaining an objective perspective.

The strengths of this study included the very high response rate, with virtually all members of the target population agreeing to participate. Also, the fact that the students’ empathy level was measured at the beginning and end of the rotation – rather than immediately before and after the intervention avoiding the bias of the direct influence of either workshop. However, it is also a limitation of the study as it is difficult to determine if changes are a result of the workshops or due to other experiences encountered over the course of the rotation. Additionally, it is impossible to isolate the arts-making components of the activity from its context within a facilitative environment that included directions and discussion. Any changes observed in the arts-making workshop may be a result of the art or may be due to the manner in which the arts were offered. The results of the qualitative aspect of the study were strengthened by the multi-modal format of the qualitative data. Asking students to write narratives, compose poetry and make art around a single trigger allowed for complementary means of reflection which could be triangulated to better evaluate students’ empathic understanding. Potential researcher bias and subjectivity were minimized by independent coding. However, the small sampling frame of a single cohort of students potentially limits our findings to these participants.

## Conclusion

The observation that a single, 3-hour workshop affected emotional awareness suggests a promising role for such an activity to complement related teaching, rather than serve as a stand-alone component within the clinical clerkship. Such an expanded programme may be more influential as recent research findings have shown that empathy among third year medical students can be preserved with a longitudinal educational intervention [7]. While the art-making workshop may have promoted increased emotional awareness, self-understanding is only part of being a competent doctor. A future version of the workshop could include practical skills on recognizing emotions and how to monitor them to use towards increased patient understanding, rather than dismissing them. Focusing future workshops on self-awareness as an aspect of clinical interpersonal skills training would develop holistic practice as well as encourage empathic understanding of patients.
